# 4-Benzyl-*N*-methyl­piperazine-1-carbothio­amide

**DOI:** 10.1107/S1600536812005685

**Published:** 2012-02-17

**Authors:** Amer M. Alanazi, Ali A. El-Emam, Nasser R. El-Brollosy, Seik Weng Ng, Edward R. T. Tiekink

**Affiliations:** a Department of Pharmaceutical Chemistry, College of Pharmacy, King Saud University, Riyadh 11451, Saudi Arabia; b Department of Chemistry, University of Malaya, 50603 Kuala Lumpur, Malaysia

## Abstract

The asymmetric unit in the title thio­urea derivative, C_13_H_19_N_3_S, comprises three independent mol­ecules (*A*, *B* and *C*). The thio­urea groups are superimposable for the three mol­ecules, but there are significant conformational differences. Mol­ecules *A* and *B* are approximate mirror images of each other, and mol­ecule *C* has an inter­mediate conformation. The dihedral angles between the thio­urea groups and the phenyl rings are 52.10 (5), 63.29 (5) and 66.46 (6)° in mol­ecules *A*, *B* and *C*, respectively. Each independent mol­ecule self-associates into a supra­molecular chain along [100] *via* N—H⋯S hydrogen bonds. Mol­ecules of *A* and *B* assemble into layers four mol­ecules thick in the *ac* plane *via* C—H⋯S and C—H⋯π inter­actions. Mol­ecules of *C* self-assemble into layers in the *ac* plane *via* C—H⋯S inter­actions. The layers stack along the *b* axis with no specific inter­actions between them.

## Related literature

For the various biological activities exhibited by 1,4-disubstituted piperazine derivatives, see: Kadi *et al.* (2010 ▶); Al Hussainy *et al.* (2011 ▶); Moussa *et al.* (2011 ▶); Kamiński *et al.* (2011 ▶); Sheng *et al.* (2011 ▶); Yang *et al.* (2011 ▶); Liu *et al.* (2011 ▶).
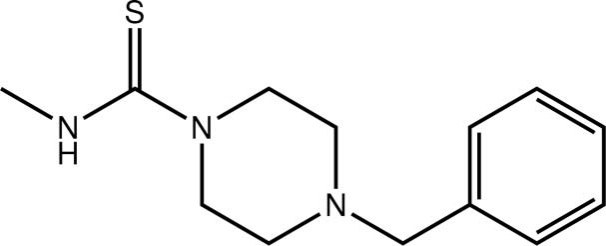


## Experimental

### 

#### Crystal data

C_13_H_19_N_3_S
*M* *_r_* = 249.37
Monoclinic, 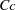

*a* = 5.8472 (1) Å
*b* = 80.3936 (9) Å
*c* = 8.6219 (1) Å
β = 103.292 (1)°
*V* = 3944.39 (9) Å^3^
*Z* = 12
Cu *K*α radiation
μ = 2.03 mm^−1^
*T* = 100 K
0.40 × 0.30 × 0.20 mm

#### Data collection

Agilent SuperNova Dual diffractometer with an Atlas detector
Absorption correction: multi-scan (*CrysAlis PRO*; Agilent, 2011 ▶) *T*_min_ = 0.497, *T*_max_ = 0.687
45632 measured reflections
7865 independent reflections
7864 reflections with *I* > 2σ(*I*)
*R*_int_ = 0.023

#### Refinement

*R*[*F* ^2^ > 2σ(*F* ^2^)] = 0.032
*wR*(*F* ^2^) = 0.087
*S* = 1.03
7865 reflections
475 parameters
5 restraints
H atoms treated by a mixture of independent and constrained refinement
Δρ_max_ = 0.17 e Å^−3^
Δρ_min_ = −0.28 e Å^−3^
Absolute structure: Flack (1983 ▶), 3714 Friedel pairs
Flack parameter: 0.020 (8)

### 

Data collection: *CrysAlis PRO* (Agilent, 2011 ▶); cell refinement: *CrysAlis PRO*; data reduction: *CrysAlis PRO*; program(s) used to solve structure: *SHELXS97* (Sheldrick, 2008 ▶); program(s) used to refine structure: *SHELXL97* (Sheldrick, 2008 ▶); molecular graphics: *X-SEED* (Barbour, 2001 ▶), *DIAMOND* (Brandenburg, 2006 ▶) and *Qmol* (Gans & Shalloway, 2001 ▶); software used to prepare material for publication: *publCIF* (Westrip, 2010 ▶).

## Supplementary Material

Crystal structure: contains datablock(s) global, I. DOI: 10.1107/S1600536812005685/pk2388sup1.cif

Structure factors: contains datablock(s) I. DOI: 10.1107/S1600536812005685/pk2388Isup2.hkl

Supplementary material file. DOI: 10.1107/S1600536812005685/pk2388Isup3.cml

Additional supplementary materials:  crystallographic information; 3D view; checkCIF report

## Figures and Tables

**Table 1:** Hydrogen-bond geometry (Å, °) *Cg*1 is the centroid of the C21–C26 ring.

*D*—H⋯*A*	*D*—H	H⋯*A*	*D*⋯*A*	*D*—H⋯*A*
N1—H1⋯S1^i^	0.87 (1)	2.59 (2)	3.387 (2)	153 (2)
N4—H4⋯S2^i^	0.88 (1)	2.64 (2)	3.367 (2)	140 (2)
N7—H7⋯S3^ii^	0.87 (1)	2.65 (2)	3.397 (2)	144 (2)
C3—H3b⋯S1^iii^	0.99	2.83	3.8213 (17)	175
C22—H22⋯S2^iv^	0.95	2.87	3.7867 (17)	163
C29—H29b⋯S3^v^	0.99	2.86	3.8007 (17)	160
C10—H10⋯*Cg*1^vi^	0.95	2.64	3.5665 (18)	164

Symmetry codes: (i) 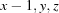; (ii) 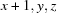; (iii) 
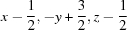
; (iv) 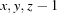; (v) 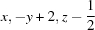; (vi) 
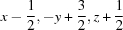
.

**Fig. 1.**
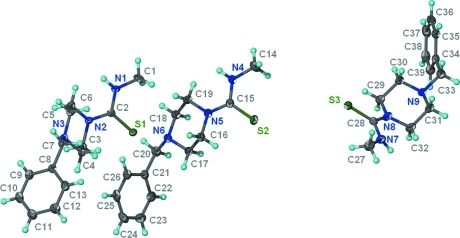
The three independent molecules of (I) showing displacement ellipsoids at the 70% probability level.

**Fig. 2.**
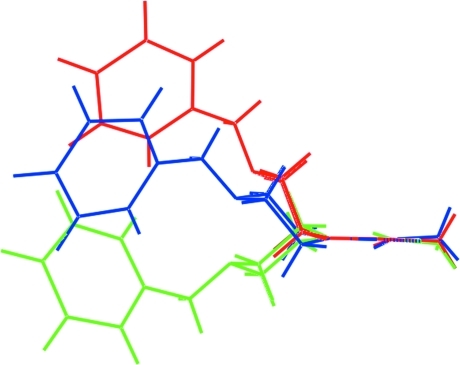
An overlay diagram of the three independent molecules in (I). The S1-, S2- and S3-containing molecules are shown as red, green and blue images, respectively. The diagram was drawn so that the thiourea SN2 planes were superimposed.

**Fig. 3.**
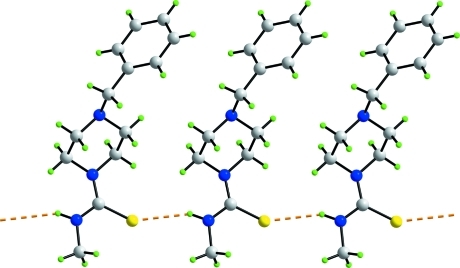
A view of the linear supramolecular chain along [100] in (I) for the S1-containing molecule. The N—H···S hydrogen bonds are shown as orange dashed lines.

**Fig. 4.**
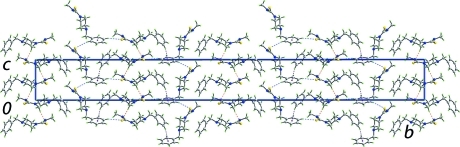
A view in projection down the a axis of the unit-cell contents for (I). The N—H···S, C—H···S and C—H···π interactions are shown as orange, blue and purple dashed lines, respectively.

## supplementary crystallographic information

### Comment

1,4-Disubstituted piperazine derivatives are known for their diverse biological activities such as a *CNS* stimulant (Al Hussainy *et al.*, 2011; Moussa *et al.*, 2011), anti-convulsant (Kamiński *et al.*, 2011), anti-microbial (Sheng *et al.*, 2011), anti-cancer (Yang *et al.* 2011) and histamine antagonist (Liu *et al.*, 2011). In continuation of our interest in the chemical and pharmacological properties of 1,4-piperazine derivatives (Kadi *et al.*, 2010), we synthesized the title compound, (I), as an intermediate for potential chemotherapeutic agents.

Three independent molecules comprise the asymmetric unit of (I), Fig. 1. There are significant differences in conformation between these as highlighted in Fig. 2. The independent molecules containing the S1 and S2 are approximately mirror images of each other and the conformation of the S3 containing molecule is intermediate between those of the others. The dihedral angles formed between the thiourea moiety and the phenyl rings are 52.10 (5), 63.29 (5) and 66.46 (6)°, respectively. Each piperazine ring has a chair conformation.

Each independent molecule self-associates into a supramolecular chain *via* N—H···S hydrogen bonds, Table 1. Chains are orientated along the *a* axis and an example is illustrated for the S1-containing molecule in Fig. 3. In the crystal packing, the S1- and S2-containing chains are connected into layers four molecules thick *via* C—H···S and C—H···π interactions. The S3-containing molecules are also connected into layers *via* C—H···S interactions. Globally, layers, which are formed in the *ac* plane, stack along the *b* axis, Fig. 4, with no specific interactions between them.

### Experimental

Methyl isothiocyanate (3.66 g, 0.05 mol) was added to a solution of 1-benzylpiperazine (8.81 g, 0.05 mol) in ethanol (15 ml). The mixture was stirred for 5 min. at room temperature and allowed to stand for 1 h. The separated crude product was filtered, washed with cold ethanol, dried and crystallized from ethanol to yield 11.60 g (93%) of the title compound as colourless crystals. *M.p.*: 365–367 K.

### Refinement

Carbon-bound H-atoms were placed in calculated positions [C—H = 0.95 to 0.98 Å, *U*_iso_(H) = 1.2 to 1.5*U*_eq_(C)] and were included in the refinement in the riding model approximation. The amino H-atoms were located in a difference Fourier map, and were refined with a distance restraint of N–H 0.88 (1) Å; their *U*_iso_ values were refined.

### Crystal data

**Table:**

C_13_H_19_N_3_S	*F*(000) = 1608
*M**_r_* = 249.37	*D*_x_ = 1.260 Mg m^−^^3^
Monoclinic, *C**c*	Cu *K*α radiation, λ = 1.54184 Å
Hall symbol: C -2yc	Cell parameters from 33030 reflections
*a* = 5.8472 (1) Å	θ = 3.3–76.0°
*b* = 80.3936 (9) Å	µ = 2.03 mm^−^^1^
*c* = 8.6219 (1) Å	*T* = 100 K
β = 103.292 (1)°	Prism, colourless
*V* = 3944.39 (9) Å^3^	0.40 × 0.30 × 0.20 mm
*Z* = 12	

### Data collection

**Table:**

Agilent SuperNova Dual diffractometer with an Atlas detector	7865 independent reflections
Radiation source: SuperNova (Cu) X-ray Source	7864 reflections with *I* > 2σ(*I*)
Mirror monochromator	*R*_int_ = 0.023
Detector resolution: 10.4041 pixels mm^-1^	θ_max_ = 76.2°, θ_min_ = 3.3°
ω scan	*h* = −7→7
Absorption correction: multi-scan (*CrysAlis PRO*; Agilent, 2011)	*k* = −100→100
*T*_min_ = 0.497, *T*_max_ = 0.687	*l* = −10→9
45632 measured reflections	

### Refinement

**Table:**

Refinement on *F*^2^	Secondary atom site location: difference Fourier map
Least-squares matrix: full	Hydrogen site location: inferred from neighbouring sites
*R*[*F*^2^ > 2σ(*F*^2^)] = 0.032	H atoms treated by a mixture of independent and constrained refinement
*wR*(*F*^2^) = 0.087	*w* = 1/[σ^2^(*F*_o_^2^) + (0.0617*P*)^2^ + 2.8424*P*] where *P* = (*F*_o_^2^ + 2*F*_c_^2^)/3
*S* = 1.03	(Δ/σ)_max_ = 0.001
7865 reflections	Δρ_max_ = 0.17 e Å^−^^3^
475 parameters	Δρ_min_ = −0.28 e Å^−^^3^
5 restraints	Absolute structure: Flack (1983), 3714 Friedel pairs
Primary atom site location: structure-invariant direct methods	Flack parameter: 0.020 (8)

### Fractional atomic coordinates and isotropic or equivalent isotropic displacement parameters (Å^2^)

**Table:**

	*x*	*y*	*z*	*U*_iso_*/*U*_eq_	
S1	0.50009 (7)	0.777950 (5)	1.00020 (5)	0.01532 (9)	
S2	0.87096 (7)	0.897767 (5)	0.78870 (6)	0.02086 (10)	
S3	1.18962 (7)	0.971979 (5)	0.91464 (5)	0.01999 (10)	
N1	0.0947 (3)	0.776304 (18)	1.08448 (19)	0.0173 (3)	
N2	0.1815 (2)	0.753280 (17)	0.94877 (18)	0.0144 (3)	
N3	0.2430 (2)	0.718795 (17)	1.03126 (17)	0.0140 (3)	
N4	0.4150 (3)	0.90408 (2)	0.7317 (2)	0.0228 (3)	
N5	0.5198 (3)	0.88474 (2)	0.5646 (2)	0.0240 (3)	
N6	0.4284 (3)	0.869454 (18)	0.25554 (19)	0.0191 (3)	
N7	1.6534 (3)	0.974994 (19)	1.0019 (2)	0.0197 (3)	
N8	1.4576 (3)	0.999011 (19)	0.9073 (2)	0.0198 (3)	
N9	1.4317 (3)	1.034283 (18)	0.94704 (19)	0.0173 (3)	
C1	0.1387 (4)	0.79283 (2)	1.1550 (2)	0.0225 (4)	
H1A	0.0044	0.7963	1.1975	0.034*	
H1B	0.1608	0.8008	1.0734	0.034*	
H1C	0.2806	0.7925	1.2415	0.034*	
C2	0.2449 (3)	0.76844 (2)	1.0126 (2)	0.0137 (3)	
C3	0.3403 (3)	0.74281 (2)	0.8827 (2)	0.0150 (3)	
H3A	0.4718	0.7496	0.8629	0.018*	
H3B	0.2548	0.7380	0.7799	0.018*	
C4	0.4367 (3)	0.72882 (2)	0.9992 (2)	0.0141 (3)	
H4A	0.5424	0.7217	0.9535	0.017*	
H4B	0.5289	0.7336	1.1001	0.017*	
C5	0.0859 (3)	0.72945 (2)	1.0969 (2)	0.0160 (3)	
H5A	0.1731	0.7344	1.1986	0.019*	
H5B	−0.0447	0.7227	1.1191	0.019*	
C6	−0.0130 (3)	0.74327 (2)	0.9801 (2)	0.0162 (3)	
H6A	−0.1043	0.7384	0.8793	0.019*	
H6B	−0.1194	0.7504	1.0254	0.019*	
C7	0.3317 (3)	0.70532 (2)	1.1436 (2)	0.0180 (3)	
H7A	0.1977	0.7001	1.1777	0.022*	
H7B	0.4381	0.7101	1.2394	0.022*	
C8	0.4621 (3)	0.69206 (2)	1.0749 (2)	0.0156 (3)	
C9	0.3552 (3)	0.68384 (2)	0.9342 (2)	0.0190 (3)	
H9	0.1998	0.6867	0.8803	0.023*	
C10	0.4738 (3)	0.67146 (2)	0.8724 (2)	0.0206 (4)	
H10	0.3994	0.6660	0.7763	0.025*	
C11	0.7005 (3)	0.66696 (2)	0.9502 (2)	0.0224 (4)	
H11	0.7811	0.6585	0.9078	0.027*	
C12	0.8083 (3)	0.67501 (3)	1.0903 (3)	0.0270 (4)	
H12	0.9631	0.6720	1.1445	0.032*	
C13	0.6897 (3)	0.68752 (2)	1.1513 (2)	0.0221 (4)	
H13	0.7653	0.6930	1.2468	0.027*	
C14	0.4563 (4)	0.91684 (3)	0.8541 (3)	0.0289 (4)	
H14A	0.3138	0.9235	0.8450	0.043*	
H14B	0.5859	0.9240	0.8406	0.043*	
H14C	0.4972	0.9116	0.9594	0.043*	
C15	0.5853 (3)	0.89524 (2)	0.6890 (2)	0.0183 (3)	
C16	0.6725 (3)	0.87163 (2)	0.5276 (2)	0.0217 (4)	
H16A	0.6189	0.8607	0.5584	0.026*	
H16B	0.8353	0.8735	0.5897	0.026*	
C17	0.6683 (3)	0.87160 (2)	0.3512 (2)	0.0197 (4)	
H17A	0.7333	0.8822	0.3222	0.024*	
H17B	0.7686	0.8625	0.3277	0.024*	
C18	0.2841 (3)	0.88317 (2)	0.2911 (2)	0.0250 (4)	
H18A	0.1219	0.8820	0.2258	0.030*	
H18B	0.3480	0.8938	0.2622	0.030*	
C19	0.2787 (3)	0.88349 (3)	0.4658 (3)	0.0288 (4)	
H19A	0.1848	0.8931	0.4871	0.035*	
H19B	0.2035	0.8732	0.4934	0.035*	
C20	0.4227 (3)	0.86947 (2)	0.0847 (2)	0.0218 (4)	
H20A	0.4999	0.8797	0.0582	0.026*	
H20B	0.2571	0.8697	0.0238	0.026*	
C21	0.5437 (3)	0.85450 (2)	0.0335 (2)	0.0174 (3)	
C22	0.7283 (3)	0.85659 (2)	−0.0422 (2)	0.0218 (4)	
H22	0.7796	0.8675	−0.0605	0.026*	
C23	0.8382 (3)	0.84281 (3)	−0.0912 (2)	0.0261 (4)	
H23	0.9624	0.8443	−0.1442	0.031*	
C24	0.7663 (4)	0.82698 (3)	−0.0626 (2)	0.0273 (4)	
H24	0.8430	0.8176	−0.0944	0.033*	
C25	0.5821 (4)	0.82472 (2)	0.0124 (2)	0.0241 (4)	
H25	0.5324	0.8138	0.0310	0.029*	
C26	0.4710 (3)	0.83840 (2)	0.0601 (2)	0.0199 (4)	
H26	0.3449	0.8368	0.1111	0.024*	
C27	1.6719 (4)	0.95786 (2)	1.0581 (3)	0.0270 (4)	
H27A	1.8369	0.9544	1.0814	0.040*	
H27B	1.5792	0.9506	0.9756	0.040*	
H27C	1.6122	0.9570	1.1551	0.040*	
C28	1.4467 (3)	0.98267 (2)	0.9419 (2)	0.0173 (3)	
C29	1.2526 (3)	1.00855 (2)	0.8240 (2)	0.0190 (4)	
H29A	1.1104	1.0015	0.8082	0.023*	
H29B	1.2740	1.0119	0.7179	0.023*	
C30	1.2203 (3)	1.02388 (2)	0.9194 (2)	0.0185 (3)	
H30A	1.0835	1.0303	0.8606	0.022*	
H30B	1.1889	1.0205	1.0229	0.022*	
C31	1.6314 (3)	1.02468 (2)	1.0357 (2)	0.0198 (3)	
H31A	1.6020	1.0213	1.1399	0.024*	
H31B	1.7745	1.0317	1.0561	0.024*	
C32	1.6704 (3)	1.00929 (2)	0.9431 (3)	0.0224 (4)	
H32A	1.7117	1.0126	0.8425	0.027*	
H32B	1.8028	1.0027	1.0065	0.027*	
C33	1.3987 (3)	1.04941 (2)	1.0354 (2)	0.0211 (4)	
H33A	1.5498	1.0555	1.0657	0.025*	
H33B	1.3520	1.0462	1.1347	0.025*	
C34	1.2144 (3)	1.06078 (2)	0.9392 (2)	0.0191 (3)	
C35	1.0308 (3)	1.06664 (2)	1.0025 (2)	0.0228 (4)	
H35	1.0206	1.0633	1.1065	0.027*	
C36	0.8622 (4)	1.07731 (3)	0.9142 (3)	0.0293 (4)	
H36	0.7379	1.0813	0.9584	0.035*	
C37	0.8752 (4)	1.08209 (2)	0.7633 (3)	0.0291 (5)	
H37	0.7604	1.0894	0.7038	0.035*	
C38	1.0559 (4)	1.07622 (2)	0.6975 (3)	0.0258 (4)	
H38	1.0638	1.0794	0.5928	0.031*	
C39	1.2246 (3)	1.06567 (2)	0.7857 (2)	0.0224 (4)	
H39	1.3484	1.0617	0.7410	0.027*	
H1	−0.051 (2)	0.7729 (3)	1.067 (3)	0.028 (6)*	
H4	0.267 (2)	0.9013 (4)	0.694 (3)	0.042 (8)*	
H7	1.782 (3)	0.9792 (3)	0.983 (3)	0.023 (6)*	

### Atomic displacement parameters (Å^2^)

**Table:**

	*U*^11^	*U*^22^	*U*^33^	*U*^12^	*U*^13^	*U*^23^
S1	0.01399 (17)	0.01385 (17)	0.0188 (2)	−0.00057 (13)	0.00511 (14)	0.00019 (14)
S2	0.01745 (19)	0.0237 (2)	0.0202 (2)	−0.00384 (15)	0.00181 (15)	−0.00261 (17)
S3	0.0180 (2)	0.01849 (19)	0.0244 (2)	−0.00296 (15)	0.00670 (16)	−0.00357 (16)
N1	0.0150 (7)	0.0164 (7)	0.0226 (8)	0.0012 (5)	0.0084 (6)	−0.0018 (6)
N2	0.0134 (6)	0.0152 (7)	0.0162 (7)	−0.0008 (5)	0.0063 (5)	−0.0001 (5)
N3	0.0135 (7)	0.0151 (6)	0.0143 (7)	0.0008 (5)	0.0050 (5)	0.0018 (5)
N4	0.0188 (7)	0.0241 (8)	0.0249 (9)	−0.0005 (6)	0.0039 (6)	−0.0082 (6)
N5	0.0162 (7)	0.0268 (8)	0.0273 (9)	0.0014 (6)	0.0016 (6)	−0.0106 (7)
N6	0.0176 (7)	0.0171 (7)	0.0220 (8)	0.0016 (6)	0.0032 (6)	−0.0040 (6)
N7	0.0180 (7)	0.0173 (7)	0.0256 (8)	−0.0002 (5)	0.0085 (6)	0.0028 (6)
N8	0.0135 (7)	0.0189 (7)	0.0268 (8)	0.0001 (5)	0.0041 (6)	0.0037 (6)
N9	0.0146 (7)	0.0184 (7)	0.0192 (7)	0.0010 (5)	0.0044 (5)	0.0015 (6)
C1	0.0272 (9)	0.0157 (8)	0.0284 (10)	0.0031 (7)	0.0143 (8)	−0.0031 (7)
C2	0.0158 (8)	0.0139 (7)	0.0107 (8)	0.0027 (6)	0.0015 (6)	0.0030 (6)
C3	0.0178 (8)	0.0160 (7)	0.0122 (8)	0.0000 (6)	0.0057 (6)	−0.0010 (6)
C4	0.0132 (7)	0.0157 (7)	0.0140 (8)	−0.0007 (6)	0.0043 (6)	−0.0009 (6)
C5	0.0153 (8)	0.0177 (8)	0.0162 (8)	−0.0010 (6)	0.0063 (6)	0.0015 (6)
C6	0.0126 (7)	0.0160 (7)	0.0203 (9)	0.0004 (6)	0.0045 (6)	0.0008 (6)
C7	0.0216 (8)	0.0180 (8)	0.0153 (8)	0.0014 (7)	0.0063 (6)	0.0033 (6)
C8	0.0185 (8)	0.0149 (7)	0.0137 (8)	−0.0006 (6)	0.0044 (6)	0.0027 (6)
C9	0.0187 (8)	0.0188 (8)	0.0174 (9)	0.0002 (6)	−0.0003 (7)	0.0023 (6)
C10	0.0266 (9)	0.0168 (8)	0.0158 (9)	−0.0019 (7)	−0.0004 (7)	0.0000 (6)
C11	0.0262 (9)	0.0181 (8)	0.0209 (10)	0.0047 (7)	0.0013 (7)	−0.0002 (7)
C12	0.0211 (9)	0.0299 (10)	0.0256 (10)	0.0090 (8)	−0.0038 (8)	−0.0047 (8)
C13	0.0202 (9)	0.0234 (8)	0.0194 (9)	0.0023 (7)	−0.0026 (7)	−0.0039 (7)
C14	0.0329 (11)	0.0267 (9)	0.0252 (11)	0.0062 (8)	0.0026 (8)	−0.0086 (8)
C15	0.0190 (8)	0.0166 (8)	0.0190 (9)	−0.0022 (6)	0.0039 (7)	0.0005 (6)
C16	0.0181 (8)	0.0208 (8)	0.0251 (10)	0.0007 (6)	0.0024 (7)	−0.0058 (7)
C17	0.0164 (8)	0.0146 (8)	0.0279 (10)	0.0006 (6)	0.0044 (7)	−0.0030 (6)
C18	0.0169 (8)	0.0250 (9)	0.0307 (11)	0.0042 (7)	0.0005 (8)	−0.0083 (8)
C19	0.0135 (8)	0.0385 (11)	0.0322 (11)	0.0017 (8)	0.0008 (8)	−0.0148 (9)
C20	0.0227 (9)	0.0188 (8)	0.0224 (9)	0.0038 (7)	0.0020 (7)	0.0012 (7)
C21	0.0177 (8)	0.0168 (8)	0.0153 (8)	0.0015 (6)	−0.0010 (6)	−0.0005 (6)
C22	0.0209 (8)	0.0234 (8)	0.0197 (9)	−0.0006 (7)	0.0016 (7)	0.0023 (7)
C23	0.0221 (9)	0.0395 (11)	0.0162 (9)	0.0040 (8)	0.0034 (7)	−0.0018 (8)
C24	0.0315 (10)	0.0285 (9)	0.0168 (9)	0.0118 (8)	−0.0052 (8)	−0.0084 (7)
C25	0.0284 (10)	0.0183 (8)	0.0202 (9)	−0.0012 (7)	−0.0053 (8)	−0.0031 (7)
C26	0.0209 (8)	0.0199 (8)	0.0174 (9)	−0.0016 (7)	0.0014 (7)	0.0003 (6)
C27	0.0230 (9)	0.0175 (9)	0.0388 (12)	0.0024 (7)	0.0038 (8)	0.0041 (7)
C28	0.0191 (8)	0.0192 (8)	0.0148 (8)	0.0000 (6)	0.0065 (6)	−0.0017 (6)
C29	0.0162 (8)	0.0195 (8)	0.0209 (9)	0.0017 (6)	0.0032 (7)	0.0030 (7)
C30	0.0144 (8)	0.0205 (8)	0.0206 (9)	0.0006 (6)	0.0041 (7)	0.0022 (7)
C31	0.0138 (8)	0.0222 (8)	0.0224 (9)	0.0001 (6)	0.0019 (7)	0.0040 (7)
C32	0.0155 (8)	0.0184 (8)	0.0338 (11)	0.0011 (6)	0.0062 (7)	0.0045 (7)
C33	0.0210 (9)	0.0215 (8)	0.0202 (9)	0.0005 (7)	0.0034 (7)	−0.0009 (7)
C34	0.0185 (8)	0.0156 (7)	0.0220 (9)	−0.0023 (6)	0.0026 (7)	−0.0023 (7)
C35	0.0204 (9)	0.0242 (8)	0.0238 (9)	−0.0008 (7)	0.0054 (7)	−0.0057 (7)
C36	0.0219 (9)	0.0279 (9)	0.0356 (12)	0.0042 (7)	0.0014 (8)	−0.0132 (8)
C37	0.0303 (10)	0.0178 (8)	0.0331 (12)	0.0042 (7)	−0.0054 (9)	−0.0054 (7)
C38	0.0310 (10)	0.0189 (8)	0.0241 (10)	−0.0033 (7)	−0.0006 (8)	0.0004 (7)
C39	0.0225 (9)	0.0197 (8)	0.0255 (10)	−0.0005 (7)	0.0063 (7)	−0.0004 (7)

### Geometric parameters (Å, º)

**Table:**

S1—C2	1.7021 (18)	C13—H13	0.9500
S2—C15	1.7058 (19)	C14—H14A	0.9800
S3—C28	1.7003 (19)	C14—H14B	0.9800
N1—C2	1.344 (2)	C14—H14C	0.9800
N1—C1	1.459 (2)	C16—C17	1.516 (3)
N1—H1	0.873 (10)	C16—H16A	0.9900
N2—C2	1.353 (2)	C16—H16B	0.9900
N2—C3	1.462 (2)	C17—H17A	0.9900
N2—C6	1.468 (2)	C17—H17B	0.9900
N3—C5	1.462 (2)	C18—C19	1.515 (3)
N3—C4	1.467 (2)	C18—H18A	0.9900
N3—C7	1.466 (2)	C18—H18B	0.9900
N4—C15	1.342 (2)	C19—H19A	0.9900
N4—C14	1.452 (2)	C19—H19B	0.9900
N4—H4	0.879 (10)	C20—C21	1.512 (2)
N5—C15	1.349 (2)	C20—H20A	0.9900
N5—C16	1.463 (2)	C20—H20B	0.9900
N5—C19	1.474 (2)	C21—C26	1.398 (2)
N6—C20	1.466 (3)	C21—C22	1.394 (3)
N6—C17	1.465 (2)	C22—C23	1.394 (3)
N6—C18	1.463 (2)	C22—H22	0.9500
N7—C28	1.350 (2)	C23—C24	1.380 (3)
N7—C27	1.456 (2)	C23—H23	0.9500
N7—H7	0.874 (10)	C24—C25	1.390 (3)
N8—C28	1.352 (2)	C24—H24	0.9500
N8—C29	1.464 (2)	C25—C26	1.387 (3)
N8—C32	1.466 (2)	C25—H25	0.9500
N9—C31	1.461 (2)	C26—H26	0.9500
N9—C30	1.465 (2)	C27—H27A	0.9800
N9—C33	1.471 (2)	C27—H27B	0.9800
C1—H1A	0.9800	C27—H27C	0.9800
C1—H1B	0.9800	C29—C30	1.518 (2)
C1—H1C	0.9800	C29—H29A	0.9900
C3—C4	1.527 (2)	C29—H29B	0.9900
C3—H3A	0.9900	C30—H30A	0.9900
C3—H3B	0.9900	C30—H30B	0.9900
C4—H4A	0.9900	C31—C32	1.518 (3)
C4—H4B	0.9900	C31—H31A	0.9900
C5—C6	1.521 (2)	C31—H31B	0.9900
C5—H5A	0.9900	C32—H32A	0.9900
C5—H5B	0.9900	C32—H32B	0.9900
C6—H6A	0.9900	C33—C34	1.508 (3)
C6—H6B	0.9900	C33—H33A	0.9900
C7—C8	1.509 (2)	C33—H33B	0.9900
C7—H7A	0.9900	C34—C35	1.393 (3)
C7—H7B	0.9900	C34—C39	1.395 (3)
C8—C13	1.391 (3)	C35—C36	1.394 (3)
C8—C9	1.397 (2)	C35—H35	0.9500
C9—C10	1.388 (3)	C36—C37	1.375 (3)
C9—H9	0.9500	C36—H36	0.9500
C10—C11	1.389 (3)	C37—C38	1.391 (3)
C10—H10	0.9500	C37—H37	0.9500
C11—C12	1.387 (3)	C38—C39	1.388 (3)
C11—H11	0.9500	C38—H38	0.9500
C12—C13	1.392 (3)	C39—H39	0.9500
C12—H12	0.9500		
			
C2—N1—C1	123.32 (15)	N6—C17—C16	110.94 (15)
C2—N1—H1	119.3 (17)	N6—C17—H17A	109.5
C1—N1—H1	115.2 (18)	C16—C17—H17A	109.5
C2—N2—C3	122.50 (14)	N6—C17—H17B	109.5
C2—N2—C6	124.76 (15)	C16—C17—H17B	109.5
C3—N2—C6	110.14 (13)	H17A—C17—H17B	108.0
C5—N3—C4	109.39 (13)	N6—C18—C19	111.49 (17)
C5—N3—C7	109.60 (13)	N6—C18—H18A	109.3
C4—N3—C7	111.04 (13)	C19—C18—H18A	109.3
C15—N4—C14	124.31 (17)	N6—C18—H18B	109.3
C15—N4—H4	120 (2)	C19—C18—H18B	109.3
C14—N4—H4	115 (2)	H18A—C18—H18B	108.0
C15—N5—C16	123.23 (16)	N5—C19—C18	109.87 (16)
C15—N5—C19	124.14 (16)	N5—C19—H19A	109.7
C16—N5—C19	112.05 (15)	C18—C19—H19A	109.7
C20—N6—C17	111.29 (15)	N5—C19—H19B	109.7
C20—N6—C18	109.20 (15)	C18—C19—H19B	109.7
C17—N6—C18	108.50 (14)	H19A—C19—H19B	108.2
C28—N7—C27	123.50 (16)	N6—C20—C21	112.84 (15)
C28—N7—H7	119.0 (17)	N6—C20—H20A	109.0
C27—N7—H7	115.4 (17)	C21—C20—H20A	109.0
C28—N8—C29	122.84 (15)	N6—C20—H20B	109.0
C28—N8—C32	125.57 (16)	C21—C20—H20B	109.0
C29—N8—C32	111.57 (14)	H20A—C20—H20B	107.8
C31—N9—C30	109.05 (14)	C26—C21—C22	119.05 (17)
C31—N9—C33	110.49 (15)	C26—C21—C20	120.58 (17)
C30—N9—C33	110.48 (14)	C22—C21—C20	120.37 (16)
N1—C1—H1A	109.5	C23—C22—C21	120.49 (18)
N1—C1—H1B	109.5	C23—C22—H22	119.8
H1A—C1—H1B	109.5	C21—C22—H22	119.8
N1—C1—H1C	109.5	C24—C23—C22	119.85 (19)
H1A—C1—H1C	109.5	C24—C23—H23	120.1
H1B—C1—H1C	109.5	C22—C23—H23	120.1
N1—C2—N2	117.68 (15)	C23—C24—C25	120.29 (18)
N1—C2—S1	119.53 (13)	C23—C24—H24	119.9
N2—C2—S1	122.76 (13)	C25—C24—H24	119.9
N2—C3—C4	109.83 (14)	C26—C25—C24	120.01 (18)
N2—C3—H3A	109.7	C26—C25—H25	120.0
C4—C3—H3A	109.7	C24—C25—H25	120.0
N2—C3—H3B	109.7	C25—C26—C21	120.32 (18)
C4—C3—H3B	109.7	C25—C26—H26	119.8
H3A—C3—H3B	108.2	C21—C26—H26	119.8
N3—C4—C3	110.15 (13)	N7—C27—H27A	109.5
N3—C4—H4A	109.6	N7—C27—H27B	109.5
C3—C4—H4A	109.6	H27A—C27—H27B	109.5
N3—C4—H4B	109.6	N7—C27—H27C	109.5
C3—C4—H4B	109.6	H27A—C27—H27C	109.5
H4A—C4—H4B	108.1	H27B—C27—H27C	109.5
N3—C5—C6	110.40 (14)	N7—C28—N8	116.46 (16)
N3—C5—H5A	109.6	N7—C28—S3	120.46 (14)
C6—C5—H5A	109.6	N8—C28—S3	123.08 (14)
N3—C5—H5B	109.6	N8—C29—C30	110.46 (15)
C6—C5—H5B	109.6	N8—C29—H29A	109.6
H5A—C5—H5B	108.1	C30—C29—H29A	109.6
N2—C6—C5	109.26 (14)	N8—C29—H29B	109.6
N2—C6—H6A	109.8	C30—C29—H29B	109.6
C5—C6—H6A	109.8	H29A—C29—H29B	108.1
N2—C6—H6B	109.8	N9—C30—C29	110.24 (15)
C5—C6—H6B	109.8	N9—C30—H30A	109.6
H6A—C6—H6B	108.3	C29—C30—H30A	109.6
N3—C7—C8	113.36 (14)	N9—C30—H30B	109.6
N3—C7—H7A	108.9	C29—C30—H30B	109.6
C8—C7—H7A	108.9	H30A—C30—H30B	108.1
N3—C7—H7B	108.9	N9—C31—C32	110.76 (16)
C8—C7—H7B	108.9	N9—C31—H31A	109.5
H7A—C7—H7B	107.7	C32—C31—H31A	109.5
C13—C8—C9	118.39 (16)	N9—C31—H31B	109.5
C13—C8—C7	120.94 (16)	C32—C31—H31B	109.5
C9—C8—C7	120.66 (16)	H31A—C31—H31B	108.1
C10—C9—C8	120.64 (17)	N8—C32—C31	110.32 (15)
C10—C9—H9	119.7	N8—C32—H32A	109.6
C8—C9—H9	119.7	C31—C32—H32A	109.6
C9—C10—C11	120.47 (17)	N8—C32—H32B	109.6
C9—C10—H10	119.8	C31—C32—H32B	109.6
C11—C10—H10	119.8	H32A—C32—H32B	108.1
C12—C11—C10	119.41 (18)	N9—C33—C34	112.23 (15)
C12—C11—H11	120.3	N9—C33—H33A	109.2
C10—C11—H11	120.3	C34—C33—H33A	109.2
C11—C12—C13	120.02 (18)	N9—C33—H33B	109.2
C11—C12—H12	120.0	C34—C33—H33B	109.2
C13—C12—H12	120.0	H33A—C33—H33B	107.9
C8—C13—C12	121.07 (17)	C35—C34—C39	118.79 (18)
C8—C13—H13	119.5	C35—C34—C33	120.46 (18)
C12—C13—H13	119.5	C39—C34—C33	120.75 (17)
N4—C14—H14A	109.5	C34—C35—C36	120.4 (2)
N4—C14—H14B	109.5	C34—C35—H35	119.8
H14A—C14—H14B	109.5	C36—C35—H35	119.8
N4—C14—H14C	109.5	C37—C36—C35	120.2 (2)
H14A—C14—H14C	109.5	C37—C36—H36	119.9
H14B—C14—H14C	109.5	C35—C36—H36	119.9
N4—C15—N5	117.16 (16)	C36—C37—C38	120.24 (19)
N4—C15—S2	120.08 (14)	C36—C37—H37	119.9
N5—C15—S2	122.76 (15)	C38—C37—H37	119.9
N5—C16—C17	110.51 (16)	C39—C38—C37	119.6 (2)
N5—C16—H16A	109.5	C39—C38—H38	120.2
C17—C16—H16A	109.5	C37—C38—H38	120.2
N5—C16—H16B	109.5	C38—C39—C34	120.79 (19)
C17—C16—H16B	109.5	C38—C39—H39	119.6
H16A—C16—H16B	108.1	C34—C39—H39	119.6
			
C1—N1—C2—N2	−178.60 (16)	C16—N5—C19—C18	−54.2 (2)
C1—N1—C2—S1	−0.4 (2)	N6—C18—C19—N5	57.3 (2)
C3—N2—C2—N1	−173.39 (15)	C17—N6—C20—C21	67.05 (19)
C6—N2—C2—N1	−13.5 (2)	C18—N6—C20—C21	−173.20 (15)
C3—N2—C2—S1	8.5 (2)	N6—C20—C21—C26	57.5 (2)
C6—N2—C2—S1	168.43 (13)	N6—C20—C21—C22	−123.02 (18)
C2—N2—C3—C4	103.74 (18)	C26—C21—C22—C23	0.3 (3)
C6—N2—C3—C4	−58.78 (18)	C20—C21—C22—C23	−179.21 (17)
C5—N3—C4—C3	−58.61 (17)	C21—C22—C23—C24	−1.0 (3)
C7—N3—C4—C3	−179.71 (14)	C22—C23—C24—C25	1.1 (3)
N2—C3—C4—N3	58.52 (18)	C23—C24—C25—C26	−0.5 (3)
C4—N3—C5—C6	59.46 (17)	C24—C25—C26—C21	−0.2 (3)
C7—N3—C5—C6	−178.57 (14)	C22—C21—C26—C25	0.3 (3)
C2—N2—C6—C5	−102.90 (18)	C20—C21—C26—C25	179.78 (16)
C3—N2—C6—C5	59.14 (18)	C27—N7—C28—N8	174.58 (18)
N3—C5—C6—N2	−59.65 (18)	C27—N7—C28—S3	−4.9 (3)
C5—N3—C7—C8	170.23 (14)	C29—N8—C28—N7	172.40 (17)
C4—N3—C7—C8	−68.80 (18)	C32—N8—C28—N7	−6.1 (3)
N3—C7—C8—C13	125.55 (18)	C29—N8—C28—S3	−8.1 (3)
N3—C7—C8—C9	−55.6 (2)	C32—N8—C28—S3	173.38 (15)
C13—C8—C9—C10	−0.1 (3)	C28—N8—C29—C30	125.94 (18)
C7—C8—C9—C10	−178.96 (16)	C32—N8—C29—C30	−55.4 (2)
C8—C9—C10—C11	0.4 (3)	C31—N9—C30—C29	−60.03 (19)
C9—C10—C11—C12	−0.2 (3)	C33—N9—C30—C29	178.35 (14)
C10—C11—C12—C13	−0.3 (3)	N8—C29—C30—N9	57.94 (19)
C9—C8—C13—C12	−0.4 (3)	C30—N9—C31—C32	59.9 (2)
C7—C8—C13—C12	178.45 (18)	C33—N9—C31—C32	−178.47 (15)
C11—C12—C13—C8	0.6 (3)	C28—N8—C32—C31	−126.48 (19)
C14—N4—C15—N5	−174.97 (19)	C29—N8—C32—C31	54.9 (2)
C14—N4—C15—S2	4.0 (3)	N9—C31—C32—N8	−57.3 (2)
C16—N5—C15—N4	−165.25 (18)	C31—N9—C33—C34	172.72 (15)
C19—N5—C15—N4	5.4 (3)	C30—N9—C33—C34	−66.52 (19)
C16—N5—C15—S2	15.8 (3)	N9—C33—C34—C35	128.33 (18)
C19—N5—C15—S2	−173.59 (16)	N9—C33—C34—C39	−51.8 (2)
C15—N5—C16—C17	−133.87 (19)	C39—C34—C35—C36	−0.6 (3)
C19—N5—C16—C17	54.5 (2)	C33—C34—C35—C36	179.21 (17)
C20—N6—C17—C16	179.77 (15)	C34—C35—C36—C37	0.3 (3)
C18—N6—C17—C16	59.61 (19)	C35—C36—C37—C38	0.4 (3)
N5—C16—C17—N6	−57.42 (19)	C36—C37—C38—C39	−0.7 (3)
C20—N6—C18—C19	178.58 (15)	C37—C38—C39—C34	0.4 (3)
C17—N6—C18—C19	−60.0 (2)	C35—C34—C39—C38	0.3 (3)
C15—N5—C19—C18	134.3 (2)	C33—C34—C39—C38	−179.54 (17)

### Hydrogen-bond geometry (Å, º)

Cg1 is the centroid of the C21–C26 ring.

**Table:**

*D*—H···*A*	*D*—H	H···*A*	*D*···*A*	*D*—H···*A*
N1—H1···S1^i^	0.87 (1)	2.59 (2)	3.387 (2)	153 (2)
N4—H4···S2^i^	0.88 (1)	2.64 (2)	3.367 (2)	140 (2)
N7—H7···S3^ii^	0.87 (1)	2.65 (2)	3.397 (2)	144 (2)
C3—H3b···S1^iii^	0.99	2.83	3.8213 (17)	175
C22—H22···S2^iv^	0.95	2.87	3.7867 (17)	163
C29—H29b···S3^v^	0.99	2.86	3.8007 (17)	160
C10—H10···*Cg*1^vi^	0.95	2.64	3.5665 (18)	164

Symmetry codes: (i) *x*−1, *y*, *z*; (ii) *x*+1, *y*, *z*; (iii) *x*−1/2, −*y*+3/2, *z*−1/2; (iv) *x*, *y*, *z*−1; (v) *x*, −*y*+2, *z*−1/2; (vi) *x*−1/2, −*y*+3/2, *z*+1/2.
